# Puerarin attenuates polystyrene (PS-MPs) induced oxidative stress, apoptosis and DNA damage in a Rat model

**DOI:** 10.1371/journal.pone.0355931

**Published:** 2026-09-08

**Authors:** Ebtesam Abdullah Alqhtani, Promy Virk, Rawan Matar Al-Harbi, Mai Elobeid, Seham Soliman Alterary, Weam Ibrahim Al-nogair, Manal Qayyadh Saad Alanazi, Ramesa Shafi Bhat, Nada M. Merghani, Dalia Fouad

**Affiliations:** 1 Department of Zoology, College of Science, King Saud University, Riyadh, Saudi Arabia; 2 Department of Chemistry, College of Science, King Saud University, Riyadh, Saudi Arabia; 3 Department of Biochemistry, College of Science, King Saud University, Riyadh, Saudi Arabia; 4 Central Research Laboratory, Vice Rectorate for Graduate Studies and Scientific Research, King Saud University, Riyadh, Saudi Arabia; Anhui University of Chinese Medicine, CHINA

## Abstract

Microplastics (MPs) are emerging pollutants that pose significant environmental and health concerns attributed to environmental persistence and potential ecotoxicity. The study assessed the protective efficacy of puerarin(Pr), a major isoflavone extracted from *Pueraria lobata* roots against microplastic toxicity in rats. Male albino rats (n = 42), were exposed to carboxylated polystyrene beads in two sizes, 1µm, and 5 µm for 28 days. Group I was the negative control. Groups III and IV received 1μm and 5 μm PS beads respectively while Group V and VI received the PS beads respectively along with a single oral dose of puerarin (10 mg/kg body weight). Microplastic intoxication with both sizes of PS-MP beads (1 µm and 5 µm) led to oxidative stress. Additionally, a significant (p ≤ 0.05) increase was also observed in the levels of pro-inflammatory cytokines and the pro-apoptotic proteins. The hepatic expression of *p53* was significantly reduced coupled with the suppression of *Nrf-2* followed by a downregulation of the anti-apoptotic protein Bcl-2. The DNA damage was observable with distinct comets on exposure to both sizes of MPs compared to the control. These results suggest that puerarin at the tested dosage alleviates oxidative stress, inflammation, apoptosis, and DNA damage in rats caused by PS-MPs. However, further investigations on different doses and extended exposure duration could establish dose-response correlation and translational significance.

## 1. Introduction

One Health perceives human, animal, and environmental health as one cohesive whole that are directly and indirectly interrelated. Every aspect of the One Health paradigm is impacted by plastic [1]. Plastic products are widely used globally, both in daily life and industry due to which the world production levels of plastic have escalated to 359 million tons(2018) from 15 million tons (1964) which is an alarming 24-fold increase [2].Global plastic manufacturing is estimated to have reached 400.3 million tons in 2022 [3].This has led to an enormous amount of environmental plastic debris resulting from consumer product disposal and industrial waste degradation [3,4].Consequently, the annual global plastic use grows at a rapid rate, and it output now exceeds 380 million tons [5,6]. Primarily, plastics are polymers with a high molecular weight combined with plasticizers, stabilizers, and other additives [7]. Plastic polymers commonly used in consumer products include polyethylene(PE), polystyrene (PS), and polyvinyl chloride(PVC) [7,8]. Among these polymers, PS is a colorless, transparent [9], highly synthesized and versatile material often used to manufacture transparent consumables such as food packaging and laboratory ware [10].

Once in the natural environment, plastic pollutants undergo weathering which subsequently results in the massive production of microplastics [11,12]. Degradation of plastic waste takes place mechanically/ chemically *via* hydrolysis and ultraviolet (UV) light, resulting in MPs(microplastics) (100 nm- 5 mm) and NPs(nano plastics) (100 nm) particles. Contrary to macro plastic debris, MPs and NPs are smaller and pervasive into multiple environmental compartments, thereby enhancing the risk of exposure and entry into the food chains [13]. These small and medium-sized polymeric materials are produced for application in industries, pharmaceuticals, and personal care products either intentionally or by the ongoing breakdown of larger plastic objects [14]. Primary microplastic, specifically those with diameters between 1 m and 5 m, are spherical and typically consist of polypropylene (PP), polystyrene (PS), or polyethylene (PE).

The research interest in the current times on toxicological effects of MP/NPs, with particular focus on PE,PS, and PVC, has been intriguing. Due to its robust tensile strength, capacity to swell, stable chemical characteristics, regeneration capabilities, broad surface area, and economic viability, PS is widely employed in a variety of goods. In addition, PS is inexpensive and chemically stable, it is widely utilized in industry. Nevertheless, there are recent concerns about human and marine health around the world due to the increasing use of polystyrene microplastics (PS-MPs) in the food chain [15]. The ability of MP/NPs to permeate biological barriers while maintaining a high surface area-to-mass ratio, coupled with their potential to bio accumulate through the food chain, has triggered research interests. Reduced body weight, early mortality, pulmonary diseases, neurotoxicity, transgenerational problems, oxidative stress, metabolic changes, ecotoxicity, immunotoxicity, and other types of dysfunctions are among the myriad adverse effects that arise from the accumulation of PS-MPs in various organs of organisms [15]. Consequently, this global crisis has been addressed by the research and regulatory communities with regard to the ecological and human health risks of MPs/NPs [12].

The expanded distribution and ubiquity of MPs has increased human exposure and the subsequent health risks [2]. The focal exposure pathway for humans is ingestion. On ingestion, the particles invade the circulatory system and can then move to the hepatic and renal systems, vital organs involved in detoxification [16].

Even though MPs and NPs are considered to be emerging pollutants of major concern to human health, toxicological effects of MPs on humans, including pathogenesis is limited. The previous studies over the past decade on the toxicity of MP/NP have been reported either *in vitro* or *in vivo* studies in animal models [7,17]. Previous research on the effects of MPs at the cellular level on human health revealed that MPs could potentially trigger an immune response, enhancing the production of cytokines and chemokines in a concentration-driven pattern [9,18].The conceivable mechanistic approach involved in MP/NPs induced toxicity has been postulated as; i) membrane disarray ii) oxidative damage, iii) immune stimulation and iv) genotoxicity [19,20].

The antioxidant system exhibits a key role in detoxification and the removal of harmful toxins from the body. Given the above facts on MP toxicity, it can be seen that oxidative stress is at the cornerstone of the toxicological effects. Inflammation and oxidative stress are two of the many variables that contribute to the etiology of MP toxicity. The human body has developed an antioxidant defense mechanism that includes metal chelation, free radical scavenging, and enzymatic activity to neutralize reactive oxygen species(ROS) in order to avoid ROS-directed oxidative damage. Furthermore, dietary antioxidants can help the body retain sufficient number of antioxidants [21]. Antioxidants can lower the ROS in the cellular system by either increasing the expression and activities of antioxidant enzymes by restricting the expression and activities of free radical-producing enzymes like xanthine oxidase (XO) [22].

Using natural products, which are a rich source of antioxidants and have been linked in multiple studies to the preclusion and treatment of chronic diseases due to their minimal side effects and significant therapeutic benefits [23]. Based on their chemical structures, phytochemicals are grouped into the following; phenols and polyphenols (e.g., quercetin, luteolin, genistein, resveratrol, and genistein), nitrogen-containing alkaloids (e.g.,berberine and sanguinarine), terpenes (e.g., betulin), and sulfur compounds (e.g., sulforaphane). Several studies especially on cancers have highlighted natural products in the modulation of oxidative stress and inflammatory disorders through their mechanistic action [23].

Therefore, natural antioxidants could aid in mitigating oxidative stress and associated apoptosis/DNA damage. A recent study investigated the ameliorative role of antioxidant supplements; lycopene, citric acid, and chlorella, against reproductive toxicity caused by MPs in African freshwater African catfish (*Clarias gariepinus*). Both lycopene and chlorella have been reported to ameliorate MP-induced reproductive dysfunctioning in fish [24]. Considering its benefits in pharmacology and healthcare as well as its affordability, low toxicity, and minimal side effects, *Puerariae lobata* is a traditional Chinese medicinal and edible plant that has garnered research interests in recent times. Several experimental investigations have demonstrated *P. lobata’s* ability to counteract oxidative stress recognizing its therapeutic efficacy in lowering blood sugar, treating tumors, and coronary diseases. *P. lobata* roots contains a type of isoflavone called puerarin(4,7-dihydroxy-8--d-glucosyliso-flavone), which is one of the most significant active ingredients and has a potent antioxidant capacity, as established by previous studies, which enables it to greatly enhance the antioxidant enzymes activity thereby combating against oxidative stress in various disease and cell damage models [25–27].Therefore, nutritional intervention of antioxidants offers a potentially curative strategy to alleviate the toxicity of inadvertent MP exposure to humans and animals in recent times, which was the cornerstone of the current investigation.

## 2. Materials and methods

### 2.1. Chemicals and kits

**Fig 1:**
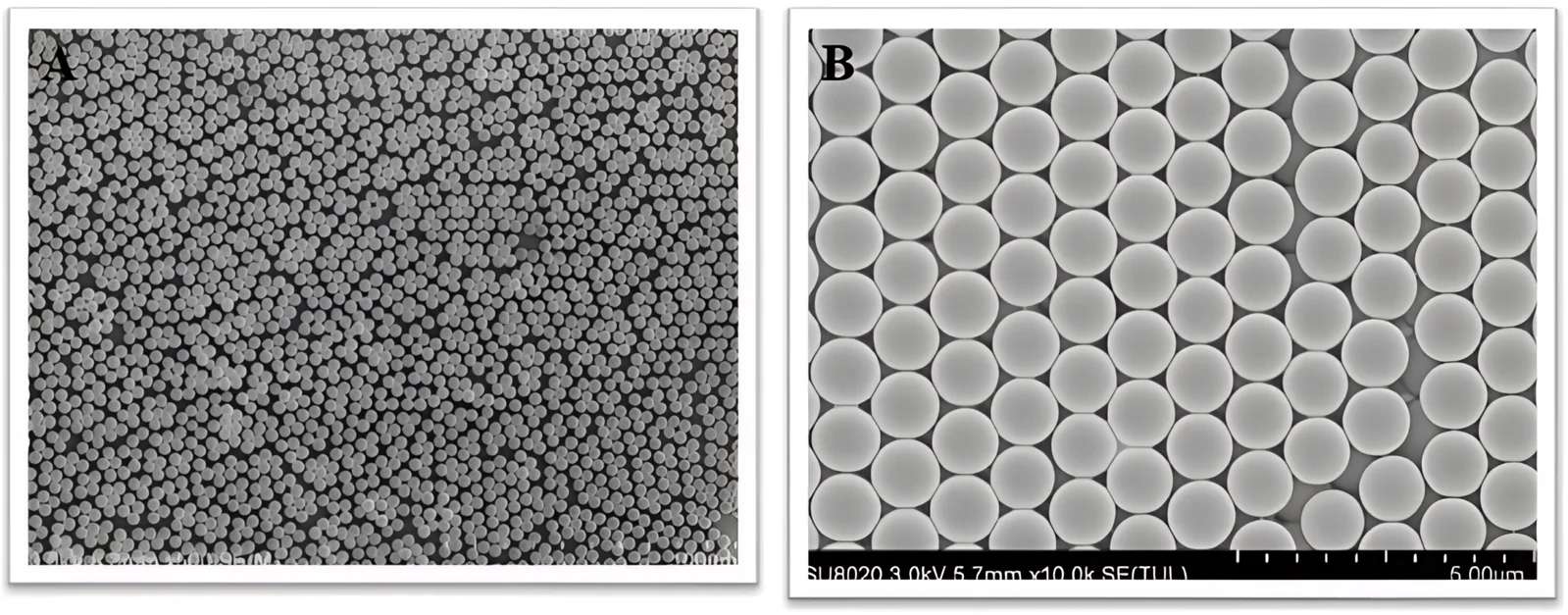
SEM micrographs (A) and (B) of PS-MPs (1 μm and 5μm) used in the study.

Ready-to-use commercial polystyrene microplastic beads(Alpha Nanotech,Canada) were used for the exposure study in two different sizes (1 and 5 μm) at a concentration of 1000 µg/L (Fig 1A and B). The PS-MPs were commercially obtained in a wet form in solution at an initial concentration is 10 mg/mL. The PS-MPs were delivered in Milli-Q-Water medium and were used as received. However, the final concentration of exposure was 1000µ/L in drinking water [2]. Polystyrene Microspheres 5 μm, Batch no. 61010 and 1µm, Batch no. 71410) (Fig 1A and B). Puerarin powder was purchased from Alibaba (Singapore). The concentration of interleukin 6, 8, 1β, (IL-6, IL-8–1β) tumor necrosis factor α (TNF-α), 8-Hydroxy-2’-deoxyguanosine (8-OHdG), malondialdehyde (MDA) levels and pro-apoptotic proteins; Caspase-3 and Caspase-6 were determined by using commercial ELISA kits (My BioSource (Inc, USA).

### 2.2. Experimental design

For the exposure study animals were housed in minimal stress conditions at the Animal House Facility at the Department of Zoology, King Saud University, Riyadh, Saudi Arabia. To ensure that distress did not exceed ethically approved severity limits, the animals were continually monitored by trained personnel hired by the institution. In addition, the study was reviewed and approved by the institutional animal care and ethics committee in accordance with the internal regulations of the local committee and sub-committees of Ethics of Research on Living Creatures issued by the Deanship of Scientific Research, King Saud University, Riyadh, Saudi Arabia(**Ethical Reference No: KSU-SE-21–51**).The Animal House Facility at King Saud University, Riyadh provided 42 adult male Albino rats, with an average weight of 150 ± 10 g. The experimental animals were acclimated in laboratory cages (7 rats/cage), at a 22 ± 2 °C maintained under a 12h photoperiod/day. Rats were given *ad libitum* regular drinking water and rodent chow. Across the rodent literature, drinking water is a frequent oral exposure route for PS-MP since drinking water is a proven human exposure source and several rodent research administer PS particles in this manner over weeks to months. Thus, in the current study the animals were exposed to the PS-MPs in drinking water. Furthermore, carboxylated PS-MPs(1 μm and 5 μm) were used in the study as carboxylation gives particles a more strongly negative, pH-responsive surface that can favor aqueous dispersion and a study has reported that zeta-potential testing showed PS-MPs had good stability and uniform water distribution [28].

The six experimental groups were as follows; Group 1: control group was given in regular drinking water. Group 2: received only puerarin (10 mg/kg b.wt) [29].Group 3: received PS MP particles (1μm) in drinking water. Group 4: received PS MP particles (5 μm) in drinking water. Group 5: received PS MP particles (1 μm) in drinking water with puerarin (10 mg/kg b.wt).Group 6: received PS MP particles (5 μm) in drinking water with puerarin (10 mg/kg b.wt).The experimental period was 28 days. During the experimental period the animals were monitored daily and the humane endpoints considered for early euthanasia were rapid/severe body weight loss, dehydration, inability to eat/drink, social withdrawal etc. However, there was no mortality observed and none of the animals reached human endpoints mentioned above.

The animals were sacrificed using the standard protocol at the Animal House Facility in the Department of Zoology at King Saud University, Riyadh, Saudi Arabia. Carbon dioxide (CO_2_) inhalation was used for euthanasia in a euthanasia glass chamber with a controlled CO_2_ flow rate of 40–60% of the chamber volume /minute. Death was confirmed by observing cessation of breathing. There were no anesthetics used in the process. Thereafter, the animals were dissected and blood samples were collected for comet assay (single-cell gel electrophoresis) and serum was prepared for the assessment of biochemical variables. Sera and tissue samples were stored at -80^o^C until analyzed further.

### 2.3. Bioassays

**2.3.1.** Biomarkers of oxidative stress (serum levels MDA and (8-OHdG), pro-inflammatory cytokines(interleukin-6;IL-6, interleukin-8; IL-8; interleukin-1β;IL-1β-, tumor necrosis factor -α;TNF-α) and apoptotic proteins(serum levels of Caspase 3 and 6) were measured in serum conforming to the instructions provided in the ELISA kit manuals. Five samples were taken from each experimental group for all the analyses.

#### 2.3.2. Quantitative real-time PCR analysis.

qRT-PCR was used to evaluate the hepatic mRNA expression of nuclear factor erythroid 2 (NFE-2) -related factor 2 (*Nrf2*) as a biomarker of oxidative stress, anti-apoptotic protein,*Bcl-2* and tumor suppressor protein, *p53* with *β-actin* serving as a housekeeping gene.The primer sequence for the target and housekeeping genes are given in Table 1.The obtained liver tissues from each experimental groups(n = 3) were minced and placed in a 2 ml homogenization tube containing a stainless steel bead sized (5 mm - 7 mm) and 600 µl of lysis buffer with 1% 2-mercaptoethanol. RNA was extracted from the homogenates using a PureLink™ RNA Mini Kit (Thermo Fisher Scientific, USA). After RNA extraction, the purity of RNA was measured using the NanoDrop2000, and the sample purity ratio 260/280 nm ranged from 1.8 to 2.0 which was further used for the cDNA synthesis. The cDNA was synthesized with the a high-capacity cDNA reverse transcription kit, Sso Advanced Universal SYBR Green Supermix (Bio-Rad, USA) using ViiA7 Real-Time PCR System(Applied Biosystems, USA).The relative quantity of mRNA was ascertained using the standard 2-ΔΔCt method.

**Table 1:** Primer sequences for target and housekeeping genes.

Gene	Reverse	Forward
***β-actin***	5- AAGGTGACAGCATTGCTTC −3’	5’- GGCCAC AATGGCTGACCATTC −3’
***Bcl-2***	5’- AGGTGCAGCTGACTGGACATCT −3’	5’- GCAGCTTCTTTCCCCGGAAGGA −3’
***p53***	5’-TTGCAGAGTGGAGGAAATGG −3’	5’- CCTATCCGGTCAGTTGTTGGA −3’
***Nrf2***	5’-CTA CAA ATG GGA ATG TCT CTGC-3’	5’-CACATCAGACAGACACCAGT-3’

#### 2.3.3. Comet assay.

An assessment of the DNA damage was based on the Comet assay in samples of whole blood collected in ethylene dinitrilo tetraacetic acid (EDTA) tubes following the modified method of Singh et al [30] and Chuang et al [31]as described previously by Virk et al [32]. The levels of DNA damage were evaluated according to five comet assay parameters, including head length(HL), tail length (TL), head intensity(HI),tail intensity (TI),and tail moment (TM).The tail intensity (%),is the fraction percentage of the genomic DNA that migrates during electrophoresis from the nuclear core to the tail. Tail and head intensities were determined automatically by image analysis software. Tail moment was calculated according to the following formula: tail moment = tail length x tail intensity/100 [33].

### 2.4. Statistical analysis

All data presented is expressed as mean ± standard error (SE) (n=5). One-way analysis of variance (ANOVA) followed by Tukey’s post hoc analysis examined the group differences using the SPSS software (ver.22; SPSS Inc., Chicago, IL, USA).A value of *p* <0.05was chosen as the threshold for the analyses, level of significance.

## 3. Results

Exposure to the tested concentrations of PS-MP beads caused no mortality in rats through the exposure period of 28 days. Also, treatment with the natural antioxidant, puerarin had no adverse effect on the experimental animals.

### 3.1. Biochemical analysis

Exposure to PS MP particles (1 µm and 5 μm) at a concentration of 1000 µg/L for 4 weeks elicited oxidative stress and associated inflammation in the experimental animals. This was evident as the serum MDA and 8-OHdG levels were significantly increased followed by enhanced levels of pro-inflammatory cytokines IL-1β, IL-6, IL-8, and TNF-ɑ. The serum levels of pro-apoptotic proteins; Caspase-3 and Caspase-6 were significantly increased. In all endpoints assessed, the controls; negative and the puerarin group showed no significant difference.

#### 3.1.1. Biomarkers of oxidative stress.

**Fig 2:**
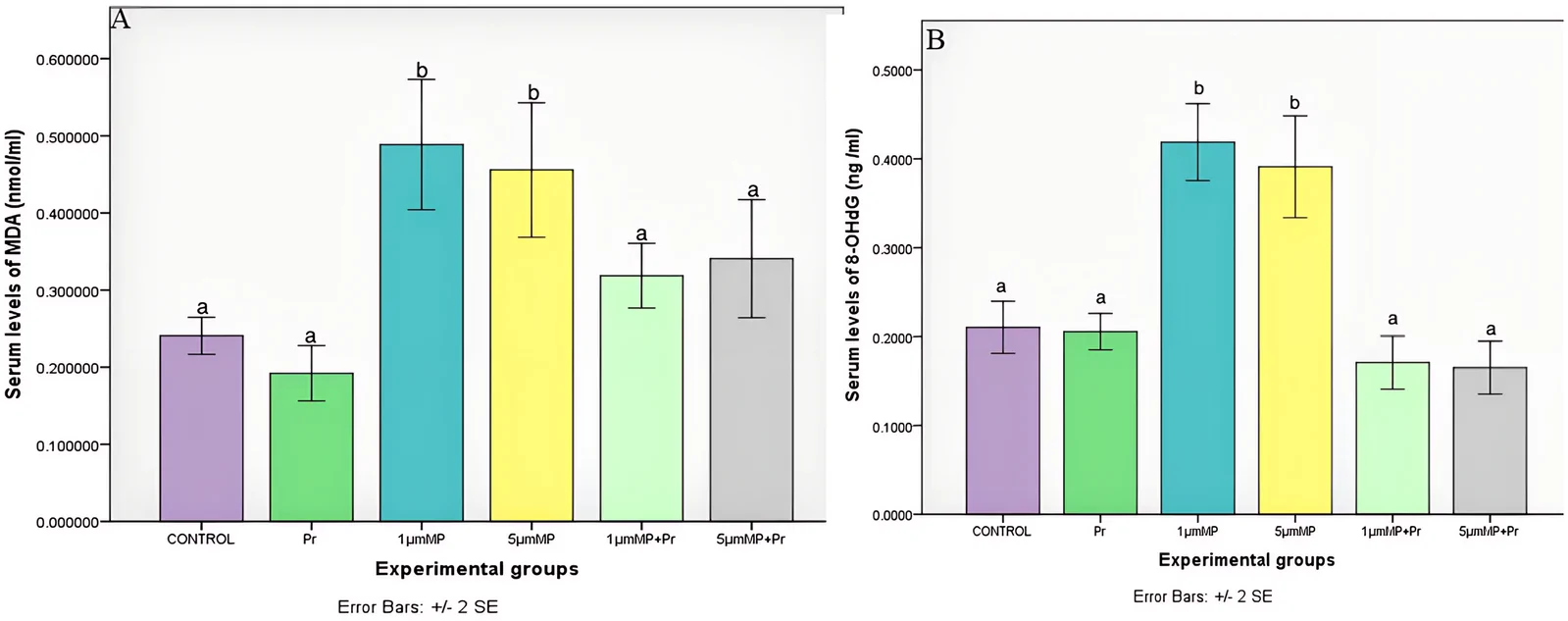
(A) Mean (±SE) serum MDA levels (nmol/ml) (B) Mean (±SE) serum 8-OHdG level (ng/ml) in rats exposed to 1 μm and 5μm MP and treated with puerarin (MP + Pr). Experimental group differences that are statistically significant (*p* ≤ 0.05) are represented by distinct letters.

The MDA levels in the serum showed a significant (p ≤ 0.05) augmentation (0.49 ± 0.42 nmol/ml) (0.46 ± 0.44 nmol/ml)on exposure to the MPs;1 μm and 5 μm respectively compared to the baseline, control(0.2407 ± 0.1204 nmol/ml). However, between 1 μm MPs group and 5 μm MPs group the difference was non-significant. MDA levels were significantly lowered on treatment with puerarin in rats exposed to 1 μm MP (0.31 ± 0.029 nmol/ml). Statistical (p ≤ 0.05) decrease was also (0.34 ± 0.03 nmol/ml) observed in the group exposed to 5 μm MP on treatment with puerarin (5 μm MP + Pr) (Fig 2A).

In addition, serum 8-OHdG levels were also significantly (p ≤ 0.05) augmented on exposure to the MPs (1 μm and 5 μm)(0.42 ± 0.02 ng/ml) and (0.39 ± 0.03 ng/ml) respectively *versus* the control (0.21 ± 0.01 ng/ml). Puerarin treatment in rats exposed to MP 1 μm significantly (p ≤ 0.05) reduced the 8-OHdG levels (0.17 ± 0.15 ng/ml). A statistical (*p* ≤ 0.05) decrease was observed in the levels (0.16 ± 0.015 ng/ml) in the group exposed to MP 5 μm on treatment with puerarin (MP 5 μm + Pr) (Fig 2B).

#### 3.1.2. Cytokines.

**Fig 3:**
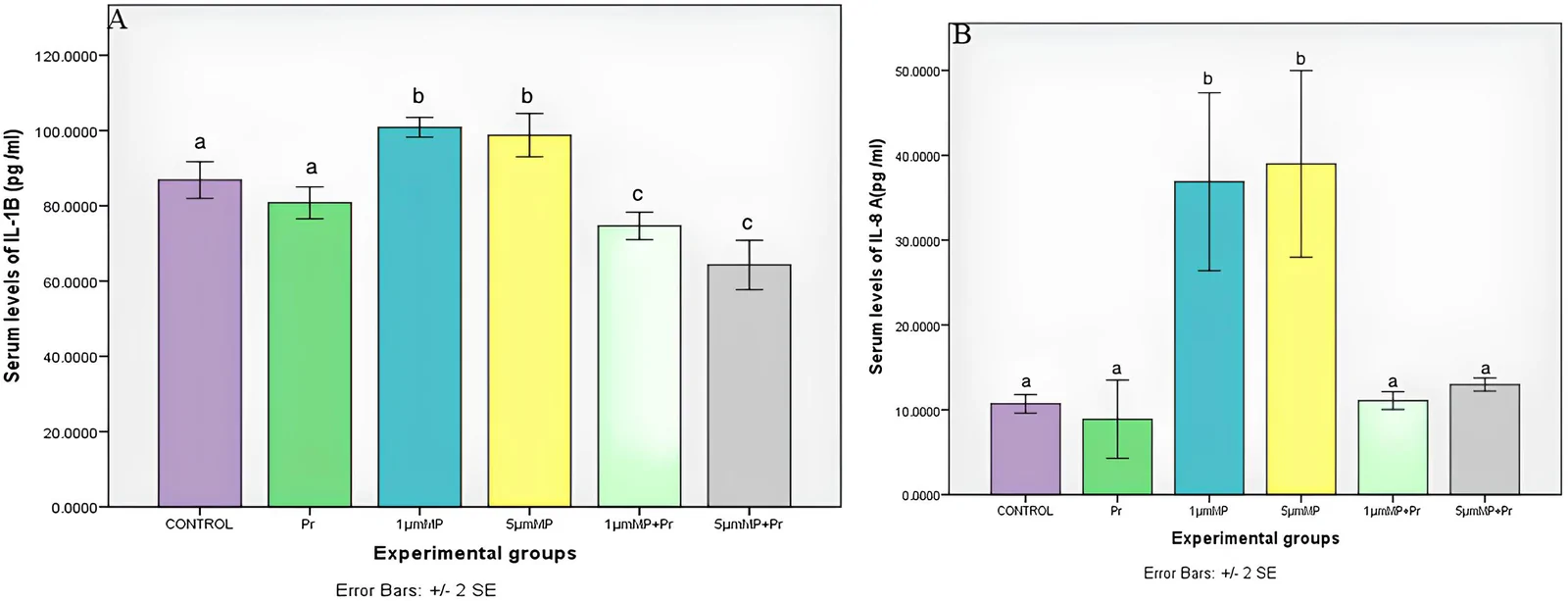
Mean (±SE) serum levels of (A) interleukin-1β (IL-1β; pg/mL) and (B) interleukin-6 (IL-6; pg/mL) in rats exposed to 1-µm and 5-µm MPs and treated with puerarin treatment (MP + Pr). Experimental group differences that are statistically significant (*p* ≤ 0.05) are represented by distinct letters.

Rats exposed to the MPs (1 µm and 5 µm) (100.83 ± 1.31 pg/ml) and (98.76 ± 2.87 pg/ml) respectively had significantly (p ≤ 0.05) higher serum IL-1 levels than controls (86.482.44 pg/ml). However, there was no discernible variance between the 1 m MPs group and the 5 μm MPs group. Treatment with puerarin resulted in a statistical (p ≤ 0.05) decrease in IL-1 levels (74.641.82 pg/ml) in rats exposed to MP at 1µm and 5µm (64.273.28 pg/ml) (Fig 3A).

In comparison to the control group (10.710.54 pg/ml), the serum levels of IL-8 were significantly (p ≤ 0.05) increased after post-exposure to the MPs; 1μm and 5μm for 28 days. The IL-8 levels between the two sizes of MPs, however, did not differ appreciably. Puerarin treatment significantly (p ≤ 0.05) decreased IL-8 levels (11.090.53 pg/ml) in rats exposed to MP 1 µm. On treatment with puerarin (MP 5 m + Pr), a substantial decrease (12.970.38 pg/ml) was also found in the group exposed to MP 5 µm (Fig 3B).

**Fig 4:**
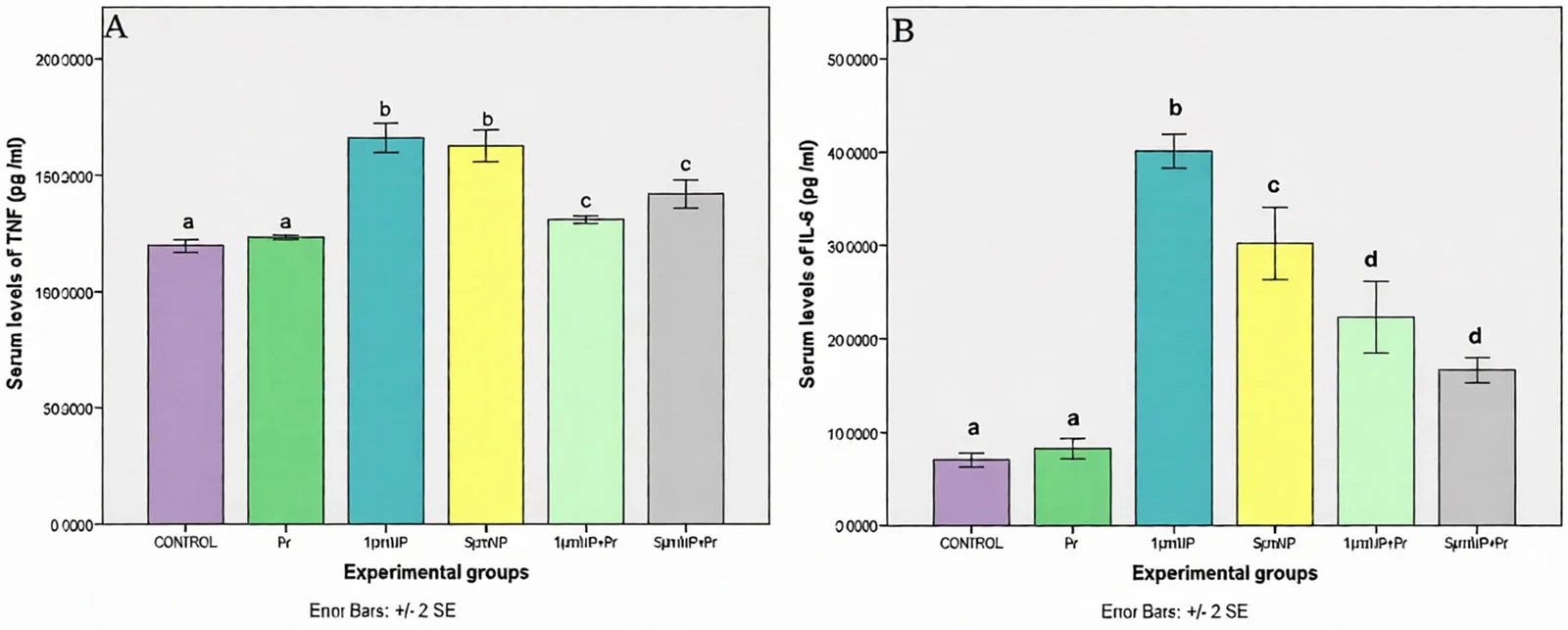
Mean (±SE) serum TNF-α (A) and IL-8 (B) levels (pg/mL) in rats exposed to 1 and 5 µm microplastics (MPs), with or without puerarin treatment (MP + Pr). Experimental group differences that are statistically significant (*p* ≤ 0.05) are represented by distinct letters.

Rats exposed to MPs at 1 μm and 5 μm had significantly(p ≤ 0.05) higher serum TNF- levels (165.94 3.08 pg/ml and (162.60 3.38 pg/ml) respectively than control (119.64 ± 1.32 pg/ml). However, there was no discernible group difference in TNF- levels was observed with 1 m MPs and 5 μm MPs. Puerarin treatment considerably decreased the TNF-levels (130.860.76 pg/ml) in rats exposed to MP 1μm. Additionally, a substantial decrease (141.913.01 pg/ml) was seen in the group, MP 5 μm + Pr (Fig 4A).

Additionally, after four weeks of exposure to the MPs; 1 μm and 5μm, the serum IL-6 levels were elevated (p ≤ 0.05) in comparison to the baseline, control (7.0063 pg/ml) to 40.13 pg/ml and 30.19 pg/ml, respectively. In contrast to the other endpoints described above, a significant difference between the groups exposed to 1 μm and 5 μm MPs was seen. Puerarin treatment also markedly reduced the IL-6 levels in rats exposed to both 1 μm MP (22.28 1.92 pg/ml) and 5 μm MP (16.620.67 pg/ml) (Fig 4B).

#### 3.1.3. Apoptotic proteins.

**Fig 5:**
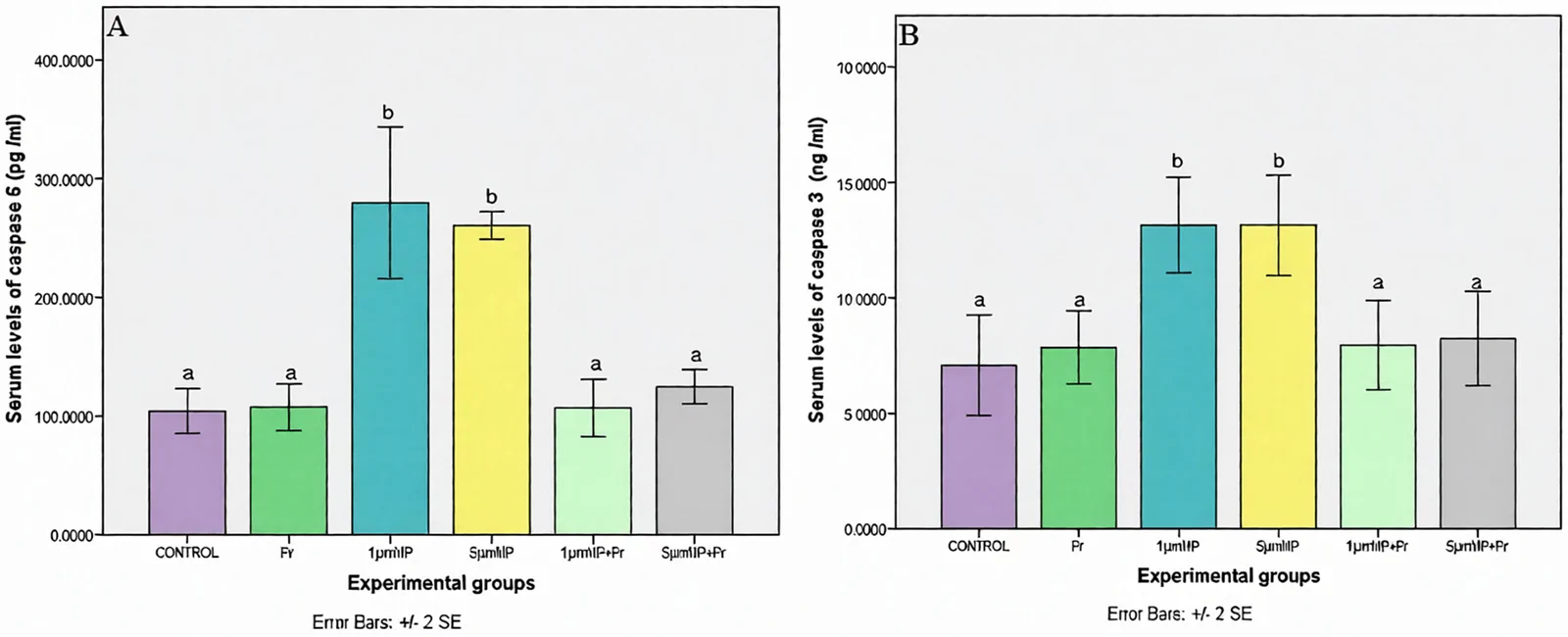
Mean (±SE) (A) serum Caspase-6 levels (ng/ml) (B) serum Caspase-3 levels (ng/ml) in rats exposed to 1 µm and 5 µm MP and treated with puerarin (MP+Pr). Experimental group differences that are statistically significant (*p* ≤ 0.05) are represented by distinct letters.

Exposure to the MPs;1 μm and 5 μm, significantly (p ≤ 0.05) augmented the serum levels of Caspase-3 levels (13.15 ± 1.04 ng/ml) (13.14 ± 1.09 ng/ml) respectively as opposed to the control (7.07 ± 1.09 ng/ml). Nevertheless, the Caspase-3 levels did not exhibit a significant difference between 1 μm MP group 5 μm MP group. Treatment with puerarin in rats exposed to MP 1 μm significantly (*p* ≤ 0.05) decreased Caspase-3 levels (7.94 ± 0.97 ng/ml). Additionally, a significant (*p* ≤ 0.05) decrease (8.23 ± 1.02 ng/ml) was also detected in the group exposed to MP 5 μm on treatment with puerarin (MP 5 μm + Pr). Additionally, exposure to the MPs; 1 μm and 5 μm also demonstrated an (*p* ≤ 0.05) elevation in Caspase-6 levels in the rats (279.42 ± 31.96 pg/ml) and (260.24 ± 5.84 pg/ml) respectively as opposed to the control (103.92 ± 9.47 pg/ml) with no significant difference among the two MP groups. Further, treatment with puerarin in rats exposed to MP 1 μm significantly (p ≤ 0.05) decreased Caspase-6 levels (106.38 ± 12.14 pg/ml). A statistical (*p* ≤ 0.05) reduction (124.32 ± 7.27 pg/ml) was also observed in rats exposed to MP 5 μm on treatment with puerarin (MP 5 μm + Pr) (Figs 5A and B).

#### 3.1.4. mRNA expression of hepatic *p53*, *Nrf-2* and *Bcl-2.*

**Fig 6:**
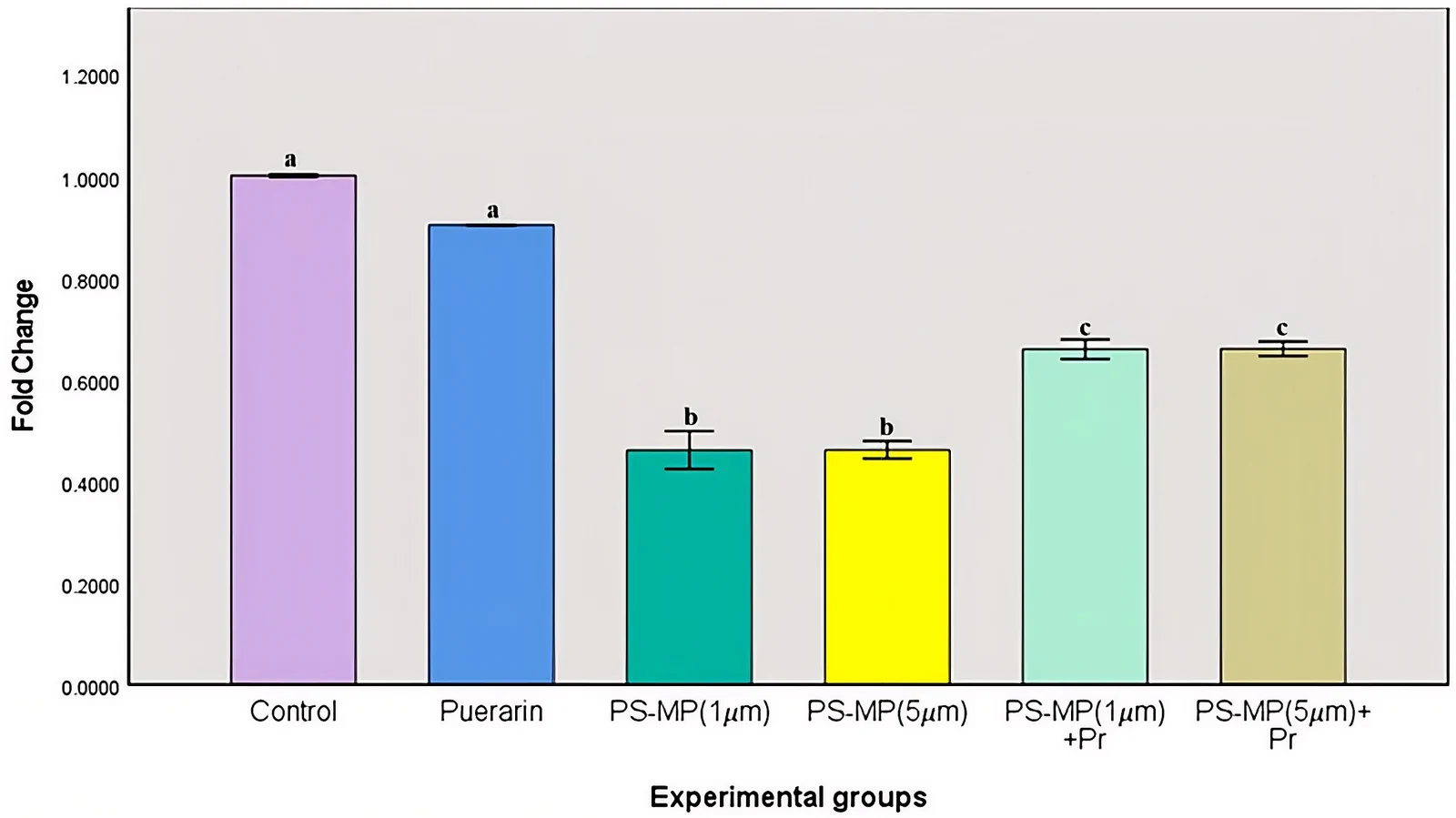
Mean (±SE) fold change in p53 mRNA expression levels in the liver of rats exposed to high dose (PS-MPs 5µm) and low dose (PS-MPs 1µm) and treated with puerarin (PS-MPs 5µm + Pr) and (PS-MPs 1µm + Pr). Different letters indicate significant (*p*  ≤  0.05) difference between the experimental groups.

**Fig 7:**
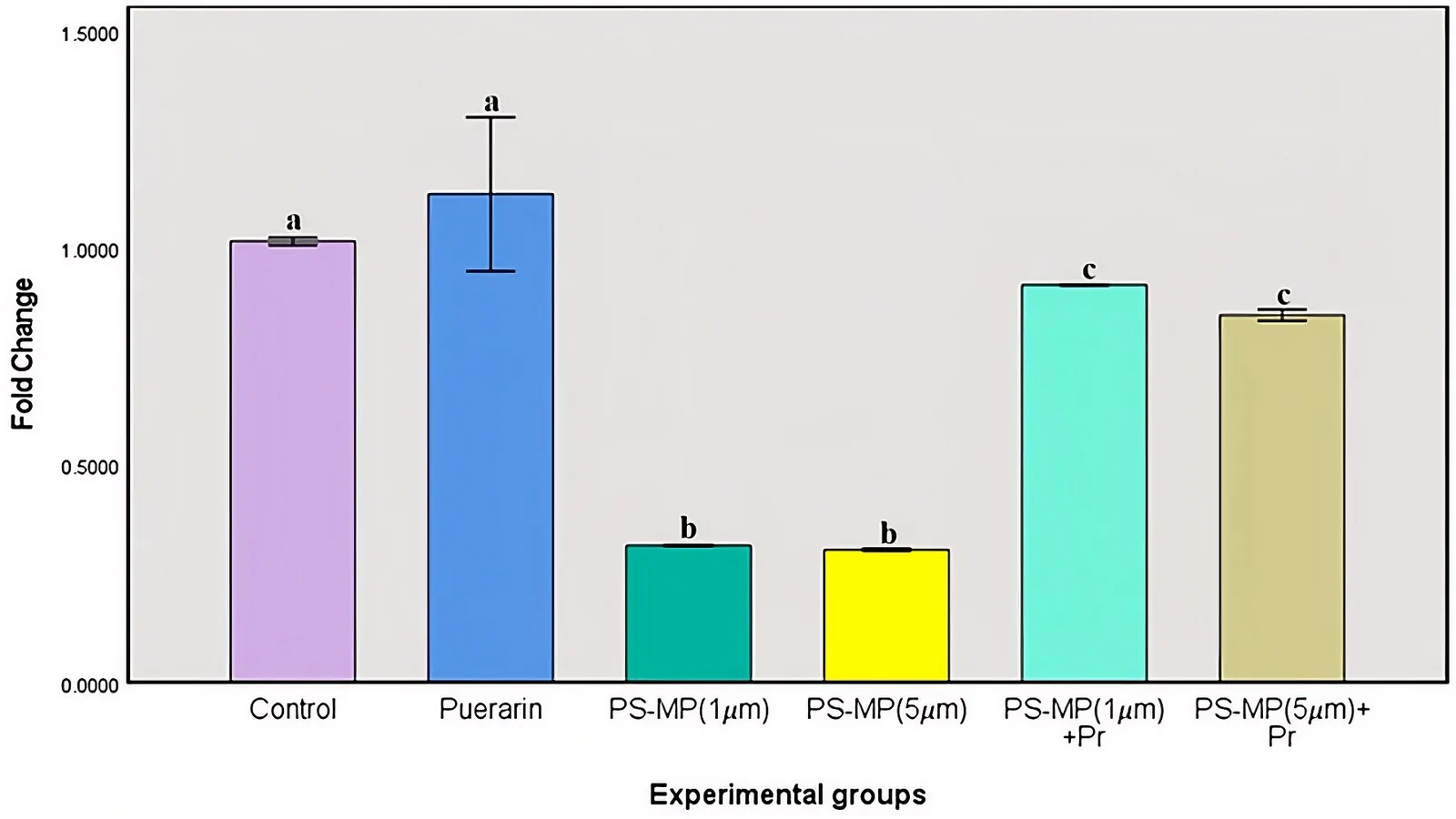
Mean (±SE) fold change in Bcl-2 expression levels in the liver of rats exposed to high dose (PS-MPs 5µm) and low dose (PS-MPs 1µm) and treated with puerarin (PS-MPs 5µm + Pr) and (PS-MPs 1µm + Pr). Different letters indicate significant (*p*  ≤  0.05) difference between the experimental groups.

**Fig 8:**
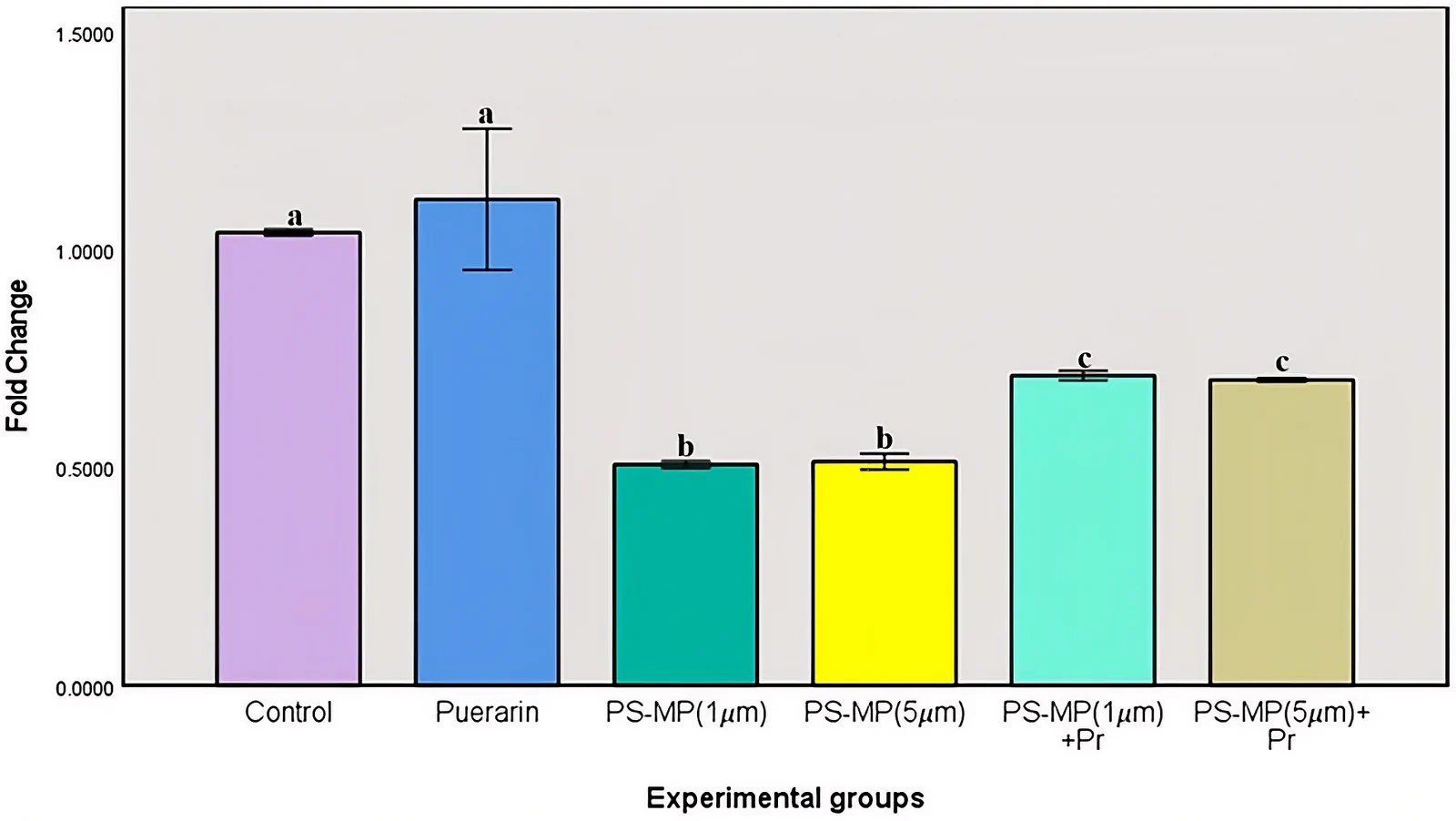
Mean (±SE) fold change in Nrf-2 mRNA expression levels in the liver of rats exposed to high dose (PS-MPs 5µm) and low dose (PS-MPs 1µm) and treated with puerarin (PS-MPs 5µm + Pr) and (PS-MPs 1µm + Pr). Different letters indicate significant (*p*  ≤  0.05) difference between the experimental groups.

Experimental exposure to PS-MPs (low and high dose) and puerarin treatment had no effect on the expression of β-actin, hence its use as a housekeeping gene was assert. When exposed to both the low and high dose of PS-MPs, the hepatic mRNA expression of the target tumor suppressor gene, p53, was considerably (p ≤  0.05) suppressed in the liver compared to the negative control(Fig 5). In addition, the mRNA expression of *Nrf-2* was also suppressed followed by a downregulation of the anti-apoptotic gene, *Bcl-2*, that resulted in apoptosis(Figs 6 and 7). However, treatment with puerarin (PS-MPs1 μm + Pr; PS-MPs 5 μm + Pr), significant (p ≤  0.05) reversed these modulatory effects on all the expression of all the three above mentioned target genes. In comparison to the negative control, the group that received only puerarin was statistically non-significant(Figs 6–8).

### 2.2. Genotoxicity

**Fig 9:**
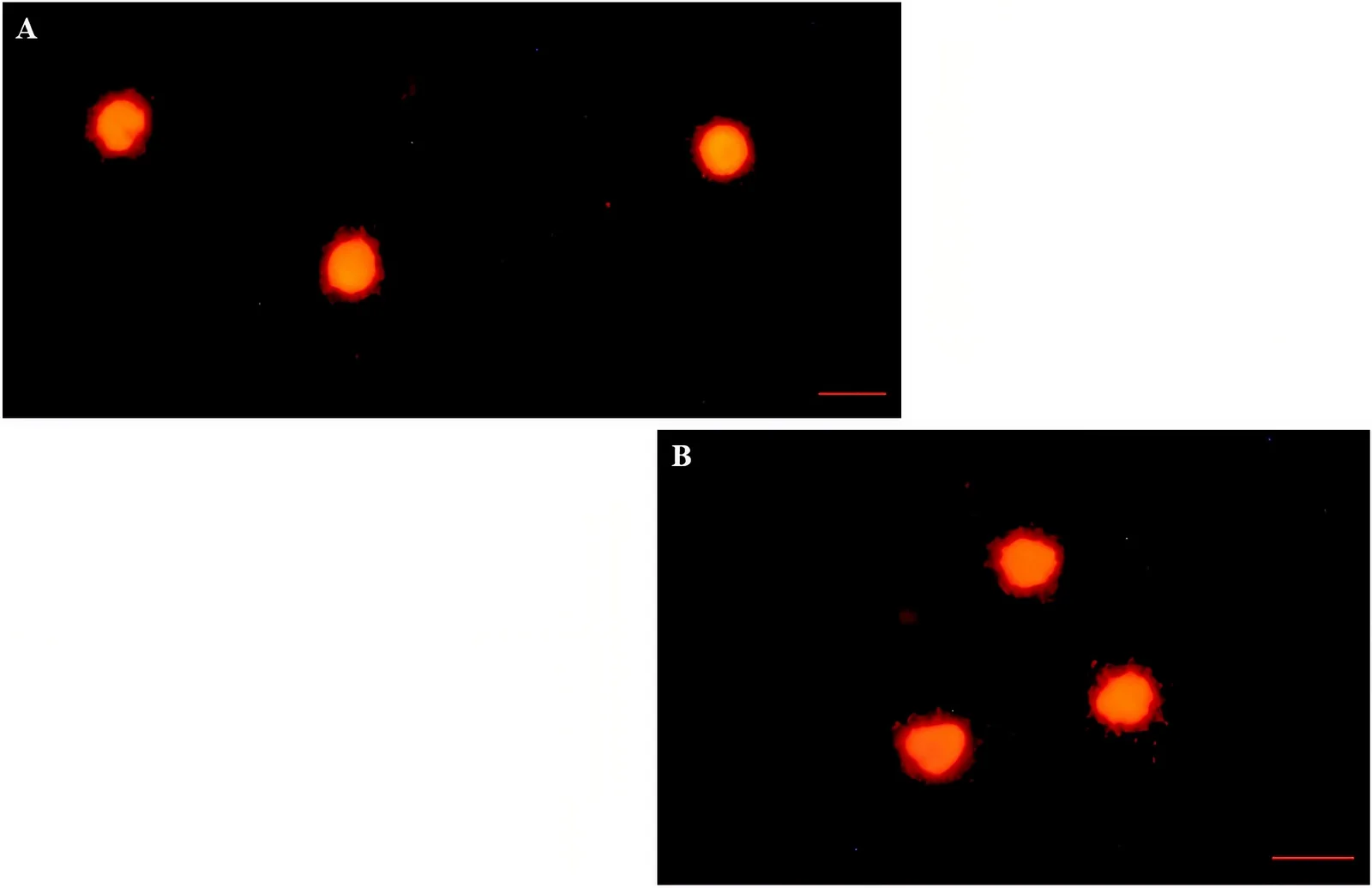
Comet images showing the effect of Pr treatment on PS-MP (1µm and 5µm) – induced DNA damage. (**A**) and (**B**) Comet images negative and positive(Puerarin) control cells;Scale bar, 50um, illustrating intact supercoiled DNA within the nuclear membrane.

**Fig 10:**
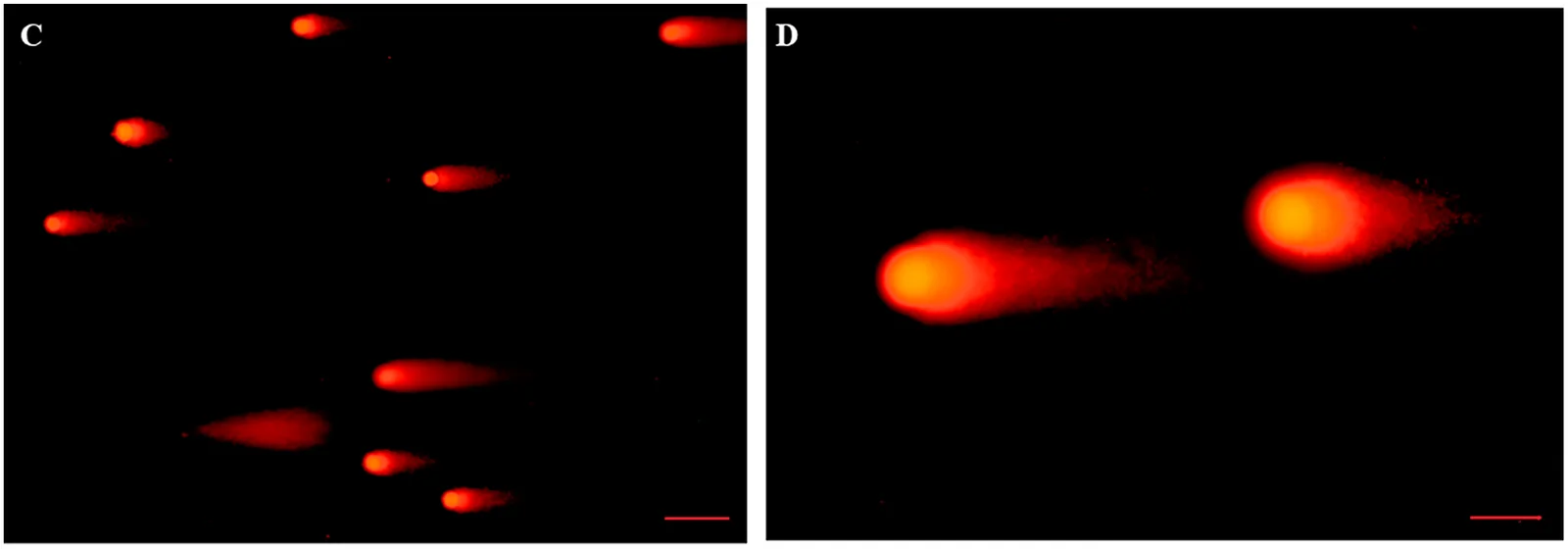
Representative comet images of cells exposed to 1 µm PS-MPs, (C) showing denatured DNA fragments characterised by migration from the nucleus, forming elongated comet tails; (D) magnified view of a representative comet (D, 40X). Scale bars: 100 µm (C) and 20 µm (D).

**Fig 11:**
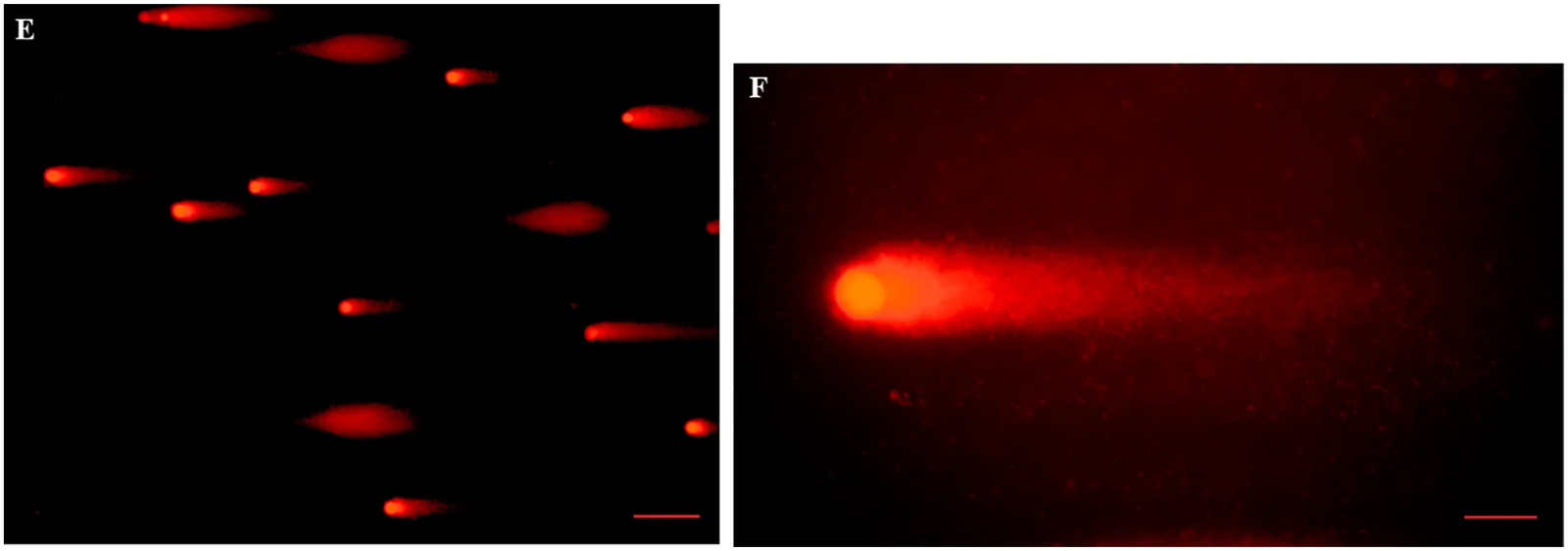
Representative comet images of cells exposed to 5 µm PS-MPs, (E) demonstrating DNA damage characterised by the migration of fragmented DNA, with elongated comet tails; (F) magnified view of a representative comet (F, 40X). Scale bars: 100 µm (E) and 20 µm (F).

**Fig 12:**
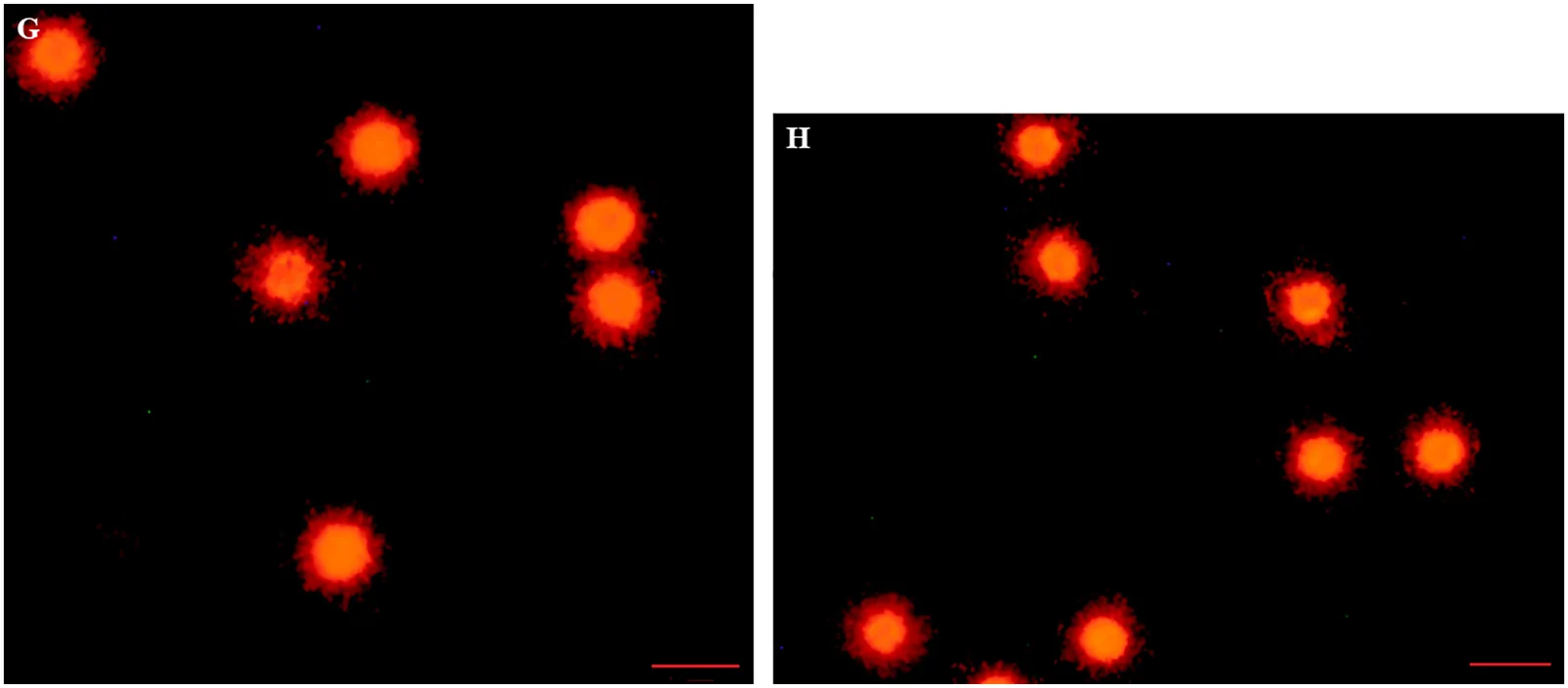
Representative comet images of cells (G) exposed to 1 µm PS-MPs and treated with puerarin (1 µm PS-MP + Pr), showing a small proportion of DNA fragments migrating from the cells compared with the time-matched controls (H) exposed to 5 µm PS-MPs and treated with puerarin (5 µm PS-MP + Pr), showing a slight degree of DNA migration compared with the corresponding controls (H). Scale bar: 50 µm.

**Fig 13:**
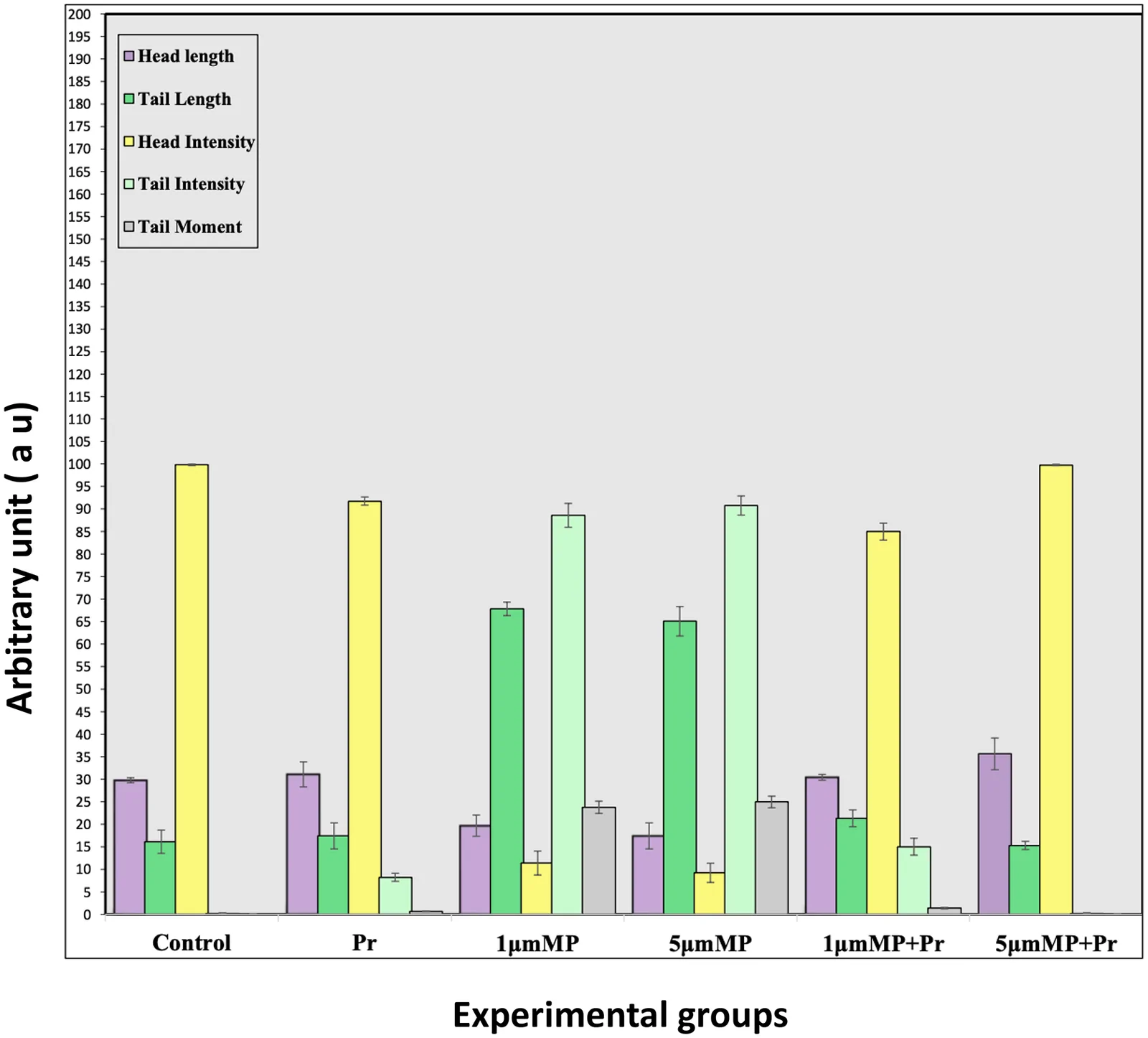
Analytical parameters of Comet assay showing the effect of Pr treatment on MP (1µm and 5µm) – induced DNA damage. Experimental group differences that are statistically significant (*p* ≤ 0.05) are represented by distinct letters.

The genotoxic potential of the MPs and the curative effect of puerarin was evaluated by the comet assay or a somatic cell genome editing in whole blood. The DNA damage in the cells was assessed based on the following parameters: HL, HI, TL, %DNA, and TM. A significant statistical (p ≤ 0.05) increase in the mean values of the TL, %DNA, and TM (67.85 ± 1.49,88.61 ± 2.65,23.77 ± 1.36, respectively) was observed on exposure to 1 µm of MP in contrast to the control sample (16.11 ± 2.583, 0.18 ± 0.16, 0.01 ± 0.01, respectively). In addition, HL and HI showed a significant (p ≤ 0.05) decrease, (19.69 ± 2.35,11.39 ± 2.66) respectively in comparison to the values in the control sample (29.77 ± 0.56, 99.82 ± 0.16). Similarly, the group exposed to 5 µm of MP also showed the formation of comets with a marked increase (p ≤ 0.05) in the mean values of the TL, %DNA, and TM (65.08 ± 3.27, 90.79 ± 2.14, 24.95 ± 1.29, respectively) with a parallel significant decrease in HL and HI (17.41 ± 2.89, 9.21 ± 2.14) in contrast to the control. However, all parameters analyzed did not exhibit any significant difference among the groups exposed to 1 µm and 5 µm (Fig 9–13).

## 4. Discussion

A plausible mechanism for cytotoxicity by MP/NPs is that uptake of these particles causes impairment of plasma/endolysosomal/nuclear membrane integrity, leading to membrane pore formation and subsequent ROS generation to mitochondria. Elevated intracellular ROS levels lead to mitochondrial damage, which subsequently triggers the release of pro apoptotic factors from mitochondria, followed by production of inflammatory cytokines, eventually activating the apoptotic pathways [33,34].The current findings illustrated that exposure to MPs in the form of carboxylated PS particles in rats for a period of 28 days led to oxidative stress which was reflected by marked alterations in the biomarkers assessed. It has been recognized that human/animal exposure to MP/NP could elicit oxidative stress, inflammatory response, immune modulation, and neurotoxicity [13,35,36]. A study by Deng et al [37] reported that exposure of mice to PS-MPs of 5 µm and 20 µm at a dose of 0.01–0.5 mg/day resulted in enhanced hepatic oxidative stress markers with bioaccumulation(gut, liver, and kidney) along with hepatic inflammation. In line with this, the current study illustrated a discernible (p ≤ 0.05) increase in LPO(lipid peroxidation) which was evident from the enhanced levels of serum MDA levels. This approach is further supported by 10% of previously reviewed studies that report lipid peroxidation and consequent deleterious effects on NP and MP exposure, suggesting that LPO as a toxicity endpoint shared by both micro-and nano-size range plastics [13]. In addition, it was observed that in the current study both sizes of MPs used augmented the serum 8-OHDG levels, a commonly used biomarker of oxidative DNA damage. In consensus with this, hepatocytes from mice incubated for 24 hours with 50 nm PS particles resulted in ROS production (increase in SOD and MDA content) with subsequent DNA damage [38]. Additionally, a study by Cheng et al. [39] on earthworms exposed to MPs for 28 days, showed enhanced levels of MDA and 8-hydroxydeoxyguanosine. Additionally, rodent experimental models exposed to PS (5–5.9 m, 0.01–1 mg day-^1^), demonstrated an elevation in ROS and MDA content paralleled with a reduced glutathione activity in the testis. Furthermore, a redox imbalance in the testis triggered production of pro-inflammatory (TNF-α, IL-1, and IL-6) and pro-apoptotic (Caspase-3) markers [24]. It has been postulated that exposure to MPs stimulates the immune function in mice, which is in consensus with the alterations in the inflammatory factors [40]. A similar study by Hou et al. [41] showed a significant increase in the levels of IL-1β and IL-1 in the group of rats exposed to 1.5 mg/kg/d group compared to the control. The results on the pro-inflammatory cytokines in the present study showed a marked increase in the serum concentrations of the cytokines assessed; IL-1β, IL-6, IL-8, and TNF-α. In consensus to this, a study by Li et al [42] reported enhanced secretion of IL-1α in serum, with the higher concentration of MPs inducing inflammatory response in the small intestine in mice exposed to PS MPs (10–150 µm). In the present study the serum levels of Caspase-3 and Caspase-6 were elevated significantly on exposure to the PS-MPs. Similar to this, a prior study revealed that TNF- and DR5 (a receptor for TNF-related apoptosis-inducing ligand) were elevated by PS-NPs in A549 human lung epithelial cells. This was accompanied by an overexpression of DR5 leading to the apoptotic proteins; caspase-8, 3, and 9. These findings imply that the apoptosis produced by MPs may involve the DR5-mediated cell death-inducing signaling pathway [7].

The above-mentioned results of the present study clearly recognize oxidative stress and apoptosis as key players in microplastic toxicity [43]. It has been established that the transcription factor Nrf2 regulates the primary cytoprotective pathway against an array of external and intracellular threats [44].The Nrf2 inactivation during oxidant-induced apoptosis observed in the present study is in congruence with previous reports by [43,45]. Furthermore, in accordance with our findings, previous studies have also reported the inactivation of the antioxidant function of Nrf2 as a prerequisite for apoptotic progression which was evident from the marked repression of the anti-apoptotic gene, Bcl-2. This is in line with previous studies that also reported decreased expression of Bcl-2 activating apoptosis with a concomitant inhibition in the expression of the Nrf2-Keap1 pathway [41]. The expression pattern of the tumor suppressor protein, p53 observed in the present study was similar to the one reported by Schimidt et al [46]where a down-regulation was observed on continued NMP exposure compared to the controls. The findings of the current study regarding Nrf2 inactivation in the setting of p53 dysfunctional response indicate the possibility of p53-independent mechanisms underpinning Nrf2 inactivation during apoptosis. This is supported by a study by Mendez-Garcıa et al [45]which showed that curcumin‑induced oxidative stress initially activates Nrf2 and its target genes, but at later times Nrf2 protein (total and nuclear) and target gene expression decline as apoptosis proceeds, even though p53 remains inactive, demonstrating p53‑independent Nrf2 inactivation during apoptosis. Nevertheless, it needs further validating studies. In addition, prolonged and overwhelming oxidative stress can disrupt p53 signaling, leading to imbalanced DNA repair and apoptotic control. This can culminate in cell death, tissue damage, and genetic instability as observed in the present study where the results on gene expression mirrored the observed results the DNA damage based on the Comet score. This is in congruence with Iqbal et al. [47] who reported that oxidative stress affects signaling pathways, which lead to carcinogenesis such as those that involve *p53, Keap1-NRF2, RB1, p21, APC*, tumor suppressor genes. Dysregulation of these processes can result in uncontrolled cell proliferation, defective DNA repair mechanisms, and avoidance of cell death, all of which are hallmarks of cancer progression.

Despite the fact that MPs/NPs can bind to or interact with DNA, there is still paucity of information on the genotoxicity [37,48]. In contrast to the intact cells without comet tails in the control group, the comet images of blood cells subjected to two different diameters of the MP (1µm and 5 µm) in the current investigation demonstrated prominent comet tails. The DNA fragmentation and migration in groups exposed to MPs was also reflected in a considerable rise in the %DNA and TM. This finding is in consensus with a study by Zheng et al [38], that showed DNA damage associated with an increase in olive TM and tail DNA percentage in rat hepatocyte suspensions exposed to PS-NPs at a level above 5 × 10^−6^ mol/L. Furthermore, ingestion of MPs (0.01, 0.1, and 1% w/w in fish food) by Japanese medaka larvae showed lowered head/body ratios, enhanced ethoxy resorufin-O-demethylase activity, and DNA breaks assessed by Comet assay [49].The current study investigations showed no significant size dependent effect of the MPs observed in the end points evaluated. Studies on the size-dependent effects of MPs on organisms are still confounding. There have been studies that have reported that a smaller size of the MP has more adverse effects than the bigger size with the same morphotype and polymer type [50,51]. Contrary to this, Fang et al. [52]reported experimental data that showed that out of the three sizes (2 µm, 10 µm and 100 µm) MPs with the median size had the highest adsorption capacity, followed by 2 µm and 100 µm MPs.

As discussed above the common denominator defining the MP toxicity is the oxidative stress triggered by ROS which subsequently enhances the inflammatory response, apoptotic pathways, and DNA damage. Thus, the antioxidant system could plausibly play a vital role in alleviating the toxicological effects of MPs. With the current trend of research moving towards the use of natural antioxidants for their therapeutic benefits, phytocompounds could be potentially used to counteract the MP toxicity in wildlife and humans. An exhaustive review by Ngum et al. [23] emphatically reported that a plethora of natural products, have been used in several studies attributed to their potent antioxidative capability their involvement in combating oxidative stress/inflammation- associated disorders. It has been widely reported that approximately 80%–90% of the global population receives primary healthcare through traditional medicine that is derived from natural materials, and approximately 73% of pharmaceutical/nutraceutical treatments are modelled with them [53]. These phytocompounds may be used to create novel therapeutic approaches owing to their potential as antioxidant, anti-inflammatory, and immunomodulatory agents.

**Fig 14:**
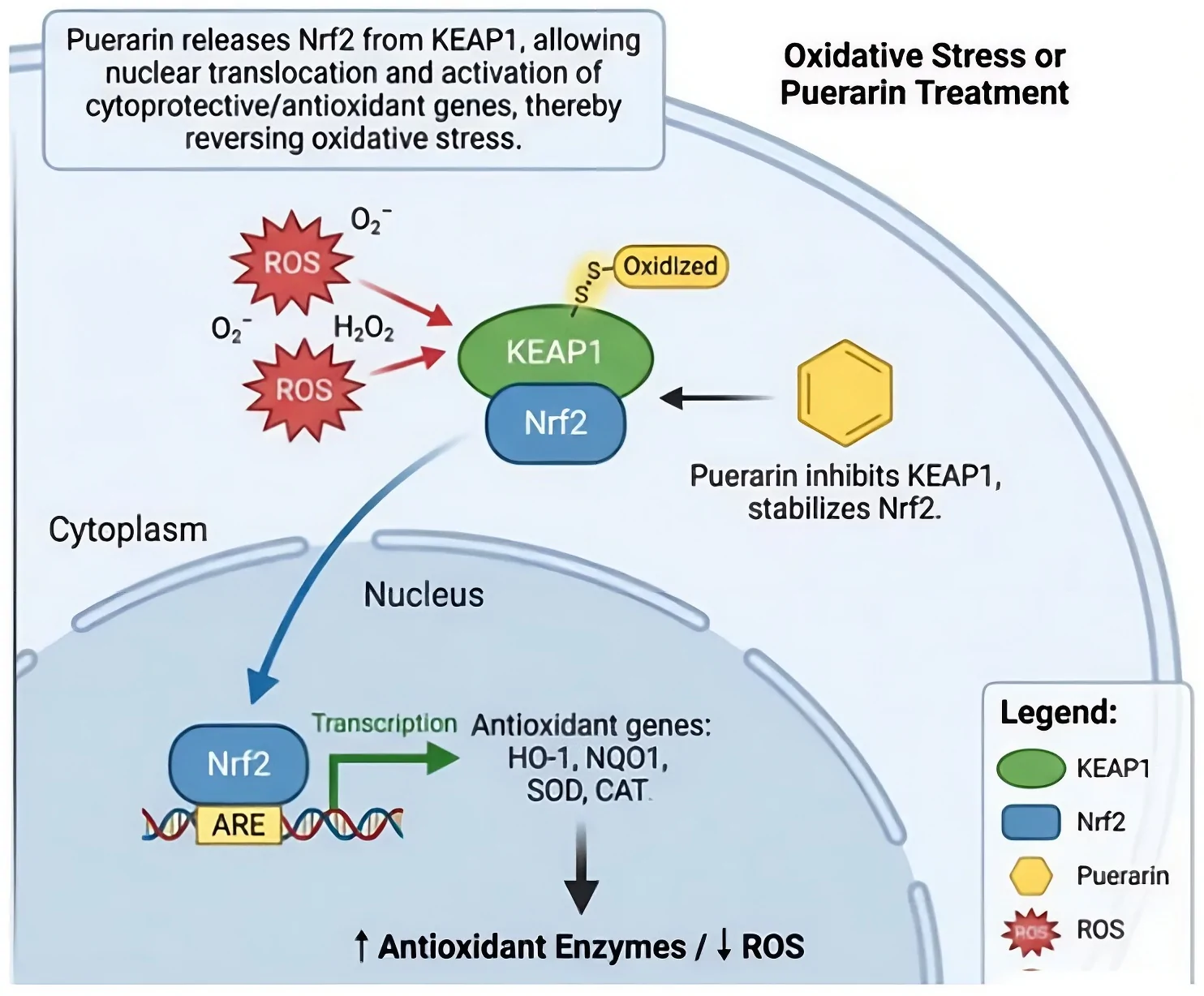
Schematic illustration of the proposed mechanistic pathway how puerarin mechanistically reverses oxidative stress.

In the current study, the co-administration of puerarin proved to be beneficial in mitigating the toxicological alterations induced by the MPs of both sizes. Treatment with puerarin reversed the modulatory effects of MP on biomarkers of oxidative stress such as serum levels of MDA and 8-OHDG, inflammatory cytokines (IL-6, IL-8, IL-1β, and TNF-α), and pro-apoptotic proteins (Caspase 3 and 6). In addition, the phytocompound showed a marked protective effect on DNA damage which was reflected by a reduction in %DNA and TM of blood cells restoring the intact cells. An exhaustive recent review on the pharmacological benefits of bioactive constituents of *Pueraria tuberosa* plant reported by Bharti et al [54] supports the findings of the current investigation. The primary bioactive component known as puerarin, used to alleviate the MP toxicity is obtained from the root of the *Pueraria lobata* plant and is one of the three main constituent bioflavonoid chemicals. Numerous studies have reported and documented its wide range of pharmacological advantages [55,56]. It has been used in the treatment of a wide range of illnesses and diseases, including diabetes/diabetic complications, Parkinson’s disease, Alzheimer’s disease, and cancer. An understanding of the mechanistic pathway through which puerarin attenuates oxidative stress shows that puerarin exerts cytoprotective effects by reactivating the *Nrf2-Keap1* antioxidant axis, restoring mitochondrial integrity *via Bcl-2* overexpression, and normalizing *p53* signaling. This coordinated control lowers oxidative stress-induced p53-independent apoptosis while restoring redox balance and controlling cellular survival. In line with this, a recent study by Mo et al [27]demonstrated the mitigating effect of puerarin on UVA radiation induced oxidative damage and photoaging in human fibroblasts. The key findings suggested that the KEAP1-Nrf2/ARE antioxidant pathway was activated after puerarin treatment, and the content and expression of antioxidant enzymes was upregulated, enhancing the overall antioxidant ability of cells to eliminate ROS and MDA generated by UVA radiation. In addition, another study by Zhang et al [57] investigated the mechanism of protective effect of puerarin *via* antioxidant function on I/R-induced neuronal injury. The experimental data suggest puerarin had a significant neuroprotective effect both *in vivo* and *in vitro*, possibly associated with the PI3K/Akt/Nrf2 signaling pathway. Puerarin treatment could activate *Nrf2 via PI3K/Akt* and enhance downstream antioxidant enzyme production, which further relieved oxidative stress and prevent I/R-induced neuronal damage. Consistent with Zhang et al.’s findings, the results of the current study support the essential involvement of the PI3K/Akt-Nrf2 axis in mediating puerarin’s cytoprotective effects under oxidative stress. In the current investigation, puerarin treatment significantly restored Nrf2 signaling. Subsequently, the observed reduction in DNA damage, lipid peroxidation, and apoptotic signaling is likely due to an increase in endogenous antioxidant capability. Overall, these findings support the molecular hypothesis that puerarin reduces toxicant-induced oxidative injury by activating PI3K/Akt-dependent Nrf2 signaling, facilitating cellular redox equilibrium and tissue protection(Fig 14). Furthermore, the oxidative stress response is characterized by a number of transcription factors that strongly control gene expression termed as OSRts; oxidative stress response transcription factors. A review in 2021 by Rizvi et al. [58] discussed how plant-derived chemicals may affect the oxidative stress response by generating structural changes in such transcription factors, particularly Nrf2 and p53. This is in light of the understanding of plant-derived chemicals such as puerarin and other antioxidants may be exploited as lead compounds to produce oxidative stress modulators.

### Limitations

Some limitations of the current study should be acknowledged. Since the major aim of this work was to provide mechanistic and therapeutic insights into microplastic-related toxicity. In this situation, using a single, established exposure dosage of puerarin and PS-MPs coupled with short experimental period (28 days) was methodologically compatible with previous experimental designs in toxicological research. Nevertheless, this may not fully reflect chronic, low-dose environmental exposure scenarios or establish dose–response relationships. Although oxidative stress, inflammation, and apoptosis were assessed through selected biochemical biomarkers and gene expression analyses, comprehensive validation at the protein level (e.g.,Western blotting, immunohistochemistry) with histopathological investigation of hepatic tissue and measurement of hepatic antioxidant enzyme activities could prove to be more robust. In addition, the study highlighted systemic toxicity and DNA damage thereby restricting organ-specific conclusions. The involvement of key signaling pathways, particularly the PI3K/Akt–Nrf2 axis, was inferred but could be mechanistically confirmed using pathway inhibitors. Finally, these findings should be interpreted within the context of the current study design, which assessed only a single dose of polystyrene microplastics and puerarin. This suggests the need for future studies to address the concerns mentioned above.

## Conclusion

The key findings of the study showed that generation of free radicals contributed towards the progression PS-MP toxicity which was evident by the modulatory effect on the biomarkers of oxidative stress, apoptosis and inflammation. In addition, DNA damage was illustrated by the comet images. Notably, puerarin therapy at the tested dose significantly reversed these changes, restoring redox equilibrium and reducing cellular damage across the tested endpoints. Overall, our findings indicate that puerarin exhibited observable protection against PS-MP-induced oxidative damage in the present experimental set up. In a larger sense, given the rising and inevitable exposure of humans and animals to microplastics, our findings indicate the possibility for dietary intervention based on nutraceuticals or phytochemical antioxidants such as puerarin as a promising prospective strategy to mitigate the toxicity of microplastics. Concurrently, the adoption of mindful plastic usage practices, tied with stringent regulatory measures to limit microplastic contamination, remains imperative for for a sustainable human existence.

## Supporting information

S1 File COMET assay images for revision. (DOCX)

S1 Data Final Raw Data supplementary data. (XLSX)
