# Supplementary material for: Puerarin attenuates polystyrene (PS-MPs) induced oxidative stress, apoptosis and DNA damage in a Rat model
Source: PLoS One. 2026 Sep 8;21(9):e0355931. doi: 10.1371/journal.pone.0355931 (PMC13552741; doi:10.1371/journal.pone.0355931)
Supplement: Supplementary file 1 [file pone.0355931.s001.docx]

**Fig.5**
